# Socioenvironmental conflicts and social representations surrounding mining extractivism at Santurban

**DOI:** 10.1038/s41598-022-14086-0

**Published:** 2022-06-15

**Authors:** Ruth Zárate-Rueda, Yolima Ivonne Beltrán-Villamizar, Daniella Murallas-Sánchez

**Affiliations:** 1 School of Social Work, Universidad Industrial de Santander, Bucaramanga, Colombia; 2 School of Education, Universidad Industrial de Santander, Bucaramanga, Colombia; 3 Centre for Technology and Innovation Management Research – INNOTEC, Universidad Industrial de Santander, Bucaramanga, Colombia

**Keywords:** Environmental sciences, Environmental social sciences

## Abstract

During the process of delimitation of the Santurban moorland ecosystem (Colombia), a socioenvironmental conflict arose from small and large-scale mining extractivism. This study analyzes the social representations of settlers in this moorland ecosystem, regarding the meaning, practice and value of their territory and water as a vital resource, in order to interpret their perceptions on the internal dynamics of conflict and the different aspects symbolizing their complexity. An ethnographic design was implemented by NVIVO software information coding. The findings underscore the uncertainty of rural stakeholders facing the impossibility to continue to develop ancestral mining activities and the disputes arising from the urban setting for the defense of water. It was concluded that the socioenvironmental conflict presented requires ongoing participation from rural and urban settlers, in consideration to their permanent questions regarding the current situation in their regions and the permanent search for solutions.

## Introduction

Mining extractivism in South America started to have an influence during the 2000s, through the extraction and export of natural resources (metals, minerals and carbon) as commodities*,* to centres of global production in Europe, North America and sectors of Asia^1,2^. In spite of experiencing a fiscal an economic crisis in the last decade, the governments of South America chose to establish extractive policies for the exploitation of mineral deposits; therefore, they persist in intensifying the extraction and superposing it to influential social and ecological aspects in the affected communities^3^.

This situation has led to the generation of socioenvironmental conflicts^4–7^. Recent studies show that in the past two decades, the South American countries with the highest incidence of socioenvironmental conflicts derived from mining extractivism are: Peru^8,9^, Chile^10,11^, Ecuador^12,13^, Argentina^14–16^, and Colombia^17,18^.

Growth in the mining sector in Colombia is linked to the production of coal, ferronickel, and gold; the latter is the second most important mineral being exploited, but it represents less royalties than ferronickel, since a large percentage of its production is illegal or informal, and the lack of operating licenses leads to deficiencies in the payment of taxes and royalties^19^. In the past decade, the Colombian government has repeatedly encouraged foreign investment, insisting on the increase of large-scale, or open-pit mining as a way to promote economic development^20,21^; hence, by February 2019, 7131 mining titles and 580 temporary authorizations had been given to natural and legal persons (21% gold/precious metals)^22^.

**Figure 1:**
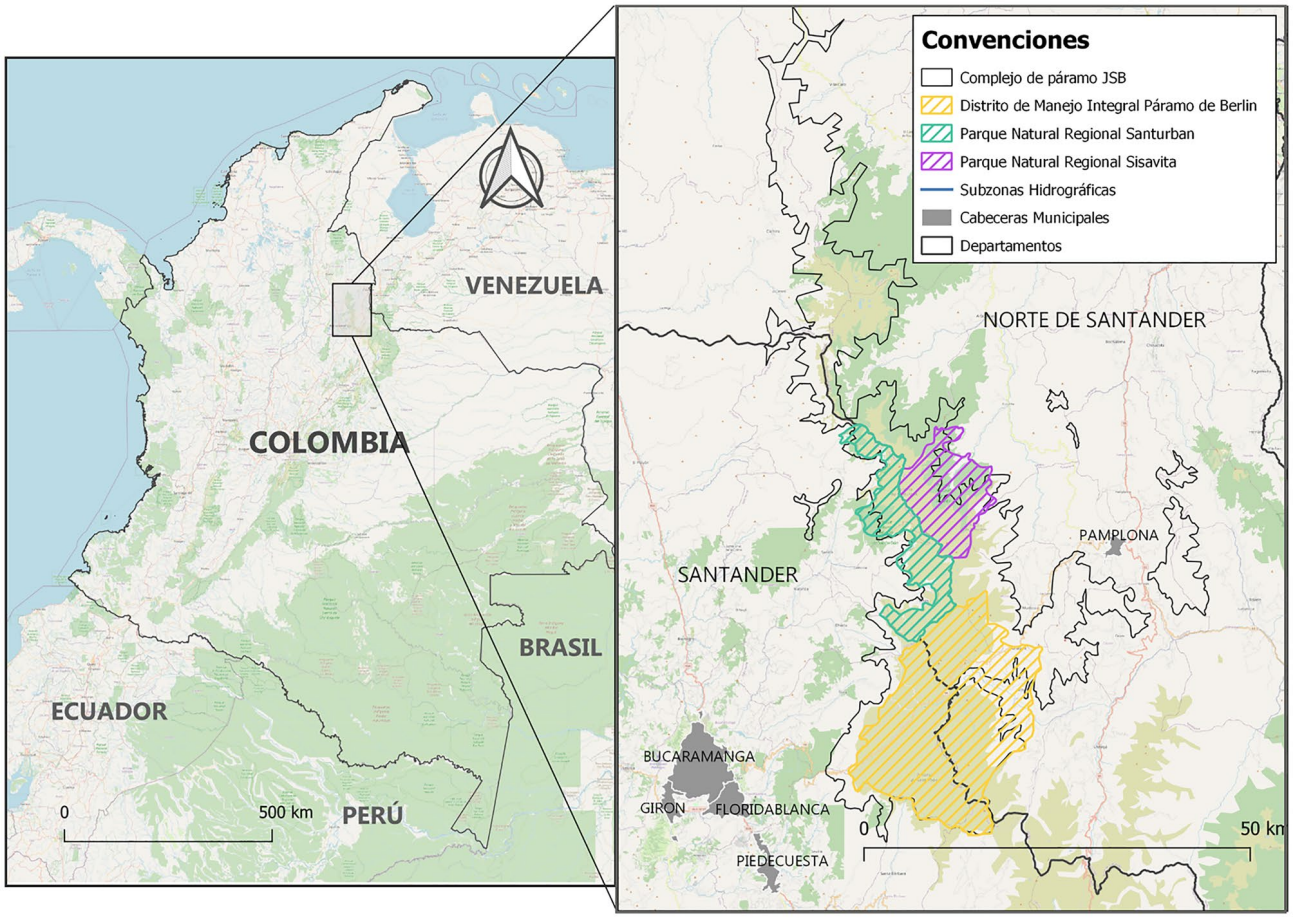
Location of the Santurban-Berlin moorland Complex, in the departments of Norte de Santander and Santander. *Source*: OPENSTREETMAP, and Sarmiento & Ungar^23^.

The Complex of Jurisdiction-Santurban-Berlin moorland ecosystem, hereinafter "the complex", is located on the eastern mountain range of Colombia, in the provinces of Norte de Santander and Santander (Fig. 1); it covers approximately 82,664 hectares, between 3000 and 4290 m above sea level (m.a.s.l.).

The moorland ecosystems are characterized by a diversity of fauna and flora, essential for socioeconomic dynamics and practices like agriculture, cattle farming and mining. They are water systems with fundamental peculiarities for the retention of water, due to the low level of the evaporation, added to the high capacity of water retention by the existing plants, and to horizontal precipitation^24^. Likewise, this Complex has been identified as an area for the exploitation of gold, especially at the municipalities of Vetas and California, since the extraction and commercialization of this material have been essential since colonial times^25^.

This area of the Colombian territory has experienced a socioenvironmental conflict caused by three differentiating factors: (1) disagreements and lack of protection of rural settlers who work in agricultural production and artisanal mining by the state; (2) socioecological damages in municipalities neighboring the moorland ecosystem who depend on its water sources and consider mining activities as a threat to the consumption of this vital liquid^26–28^. (3) An investment interest by the multinational company Sociedad Minera de Santander (Minesa) (Appendix 1), supported by the *Mubadala Investment Company* (United Arab Emirates). In the past decade, Minesa is the second company to have carried out procedures to get a license to operate in the moorland area, after the Canadian Eco Oro Minerals Corp in 2011^29^.

Against this background, in 2014, with Resolution 2090, the national government established a boundary for the Complex, in order to protect the moorland ecosystem. The boundary process is understood as the determination of the geographic limits in order to award them the title of moorland ecosystem, and thus contribute to the regulation in the use of the natural resources while conducting economic activities like cattle farming, agriculture and mining. The determination is obtained thanks to the agreement between scientific concepts and ethical knowledge that guarantee the sustainability of the moorland^30^.

The delimitation of the moorland consists of a consultation stage with the settlers of the mining area, with the purpose of defining the terms of special protection of the moorland ecosystem. It is part of the environmental management in order to guarantee ecological sustainability, in interaction with other natural environments. It should be noted that this process was defined in Sentence T-361, in response to a guardianship action presented by the people residing in the area of influence of the Santurban moorland, based on the assessment of the right to participation by the affected communities. In other words, the process of delimitation of the wasteland consists in the creation of open, wide and informed settings for communication between the peasant and urban communities and the territorial administration to carry out an analysis of the moorland boundaries that can be devoted to mining activities or be protected, either as areas devoted to agriculture, areas for ecosystem restoration in the moorland, areas adjacent to the protected areas, and river basins. By doing this, the delimitation process seeks to establish a program of productive reconversion for the traditional mining communities settled in the Santurban moorland, who are devoted to agriculture and mining, to guarantee their right to work and to a minimum income of miners who are at risk, should economic reconversion measures not be in their favour. To sum up, when authorizing mining activities in wasteland areas, and areas of restoration, the Ministry of Environment and Sustainable Development (MADS) must lead the delimitation, while guaranteeing the right to health, to drinking water and a decent living, due to the settlers’ connection with the environment, in such a way that Environmental Authorities must respect fundamental rights and the constitutional principles of environmental participation for the management of moorland ecosystems^31^.

In spite of the decision taken by the national government in 2014, this action influenced the dynamics of the socioenvironmental conflict, due to the inconsistencies related to the hectares that would be protected, the disposition of benefits to multinationals, important affectations to miners who practice Artisanal and Small-scale Mining (ASM)^32^, and the lack of participation by the communities bordering Santurban. In 2017, through Judgment T361, the Constitutional Court of Colombia lapsed the Resolution, as a result of legal actions related with the infringement of fundamental rights of participation^31^. The Colombian State must regulate the use of the natural resources, with the participation of the different stakeholders who are directly or indirectly involved with this territory. The Court allowed a term of one year to establish a new boundary; nevertheless, in January of 2022, meetings with the communities to agree on the definitive boundary are still being held.

The purpose of this study is to analyze the Social Representations (SR) of settlers in the moorland ecosystem in terms of the meanings, practices and values they hold with respect to their territory and water as vital resource. These elements are representative in a context of socioenvironmental conflict related to the risk to ecosystems and the regulation of traditional economic activities like the ASM in the moorland areas. When interpreting the perceptions of stakeholders, the internal dynamics of the socioenvironmental conflict that led to involve communities in determining the moorland boundaries is revealed. This is made evident in the conflicting perspectives between the rural inhabitants who depend on the biodiversity of the moorland area to develop ASM activities in their territories, and the urban residents as consumers of the water coming from the hydrographic river basins of the moorland.

Within the framework of the study, guiding questions were raised, such as: What are the Social Representations of the Santurban moorland in relation to mining extractivism on a small and large scale? and How can Social Representations, be interpreted with respect to the dynamics of socioenvironmental conflict, derived from ASM? To answer these questions, it was essential to apply the Theory of Social Representations from its conceptualization, since categories related to beliefs were considered from the individual to the collective, resulting from the interactions and practices of the moorland settlers in relation to the mining process. For this reason, the testimonies obtained through the collection of data correspond to the collective representations which, from a theoretical perspective, are revealed in what people think, feel and do. Hence, it was possible to achieve an understanding from inter-subjectivity and trans-subjectivity. In this regard, it is necessary to clarify that from the Theory of Social Representations the results show that Social Representations have been built in social interaction and are permeated by culture, through communicative processes that are transmitted from generation to generation and are part of the lifestyle of the moorland environment. Therefore, the theory guided the research questions and in the same way the findings supported it, because the meanings are practices of the inhabitants on the moorland ecosystem are shown, which are part of the dynamics of the socioenvironmental conflict that is generated around mining extractivism on a small and large scale.

Consequently, it was revealed that SR are defined by socially-shared knowledge^33^, together with the attitudes and attributions resulting from participation in social life^34^; hence, this paper focuses on the different edges that symbolize the complexity of a socioenvironmental conflict with transcendence in Colombia, which shows different standpoints and regulation scenarios regarding extraction, economic, social and environmental impact, community participation and the defense of the territory.

### Socioenvironmental conflicts

Conflict results from the opposing interests and expectations that originate in the integration of societies^35^, as a result of the contradiction and incompatibility arising when pursuing objectives in the exchange of values in action systems made up by individuals and groups^36,37^. Every person builds a subjective view of nature and his/her place in it, starting from conceptions, values and perceptions that make up habits and practices with respect to it^38^. Stakeholders immerse in a socioenvironmental conflict take up positions and discourse supported and legitimized through narratives related with the environment and development, defended from different approaches, and implicitly including the economic interests of transnational corporations^39^.

Reference is made to a socioenvironmental conflict, since the social dynamics is immersed in the environmental dynamics; that is, it represents a dispute between parties claiming a physical, material and immaterial space in the environmental dimension^40^. Sabatini highlights the differences between environmental and socioenvironmental conflicts. The former are related with the distribution of the external effects derived from changes in the uses of soil by new activities developed in the area; the latter are “disputes caused by the access to and control of environmental resources, especially land, but also water, minerals or others^41^”. In this sense, abusive or unequal management of natural resources leads to the degeneration of ecosystems, which breaks up stakeholders’ relationships within societies, and also involves attempts to gain control of the scarce resources^42^.

Socioenvironmental conflicts derives from environmental impact and alterations, which lead to a collective action as a public demonstration of opposition^43^. They have the following characteristics: the existence of a damage that has affected one segment of the population; the facts must be linked with the environmental dimension; the stakeholders involved assign some assessment to the damage caused, depending on the level of association with the circumstance that gives rise to the confrontation^44,45^. Finally, in an ideal setting, there is a transformation of the conflict by means of an analysis of the factors involved and the proposal for alternative solutions from the perspective of the communities affected^46^.

According to Paz, in socioenvironmental conflicts “the controversy is woven on the material and symbolic dimension of the environment; it is built around its ownerships, access and use, but also on its social construction^47^, in economic, political, juridical and cultural fields that have an impact on the local societies affected. Consequently, disputes comprise categories like: participation, modalities of land tenure, regulation in the use and control of natural resources, policies and projects in areas of conflict, the State, interests of stakeholders involved and their needs^48^. Likewise, they integrate a diversity of languages, knowledge, sensitivity and meanings^49^ which have an impact on the dynamics of conflict with discourse constructions made from intersubjectivity^50^, ontology^51^, and the modes of social cohesion to attain a collective goal^52^.

### Socioenvironmental conflicts derived from mining extractivism in South America

South America is characterized by the complexity of its socioenvironmental conflicts related to its biodiversity and dependency of its agricultural and extractive systems^53^. In the past decade, these conflicts have become made more visible and more frequent due to the prioritization of environmental aspects at global scale, the strengthening of social movements, the development of projects without prior consultation, and the active role of communities^6^. The scientific literature defines intrinsic aspects of socioenvironmental conflicts in this segment of the American continent, which stand out due to the mismatch between the increasing social demands and the negligence of governments to respond to them^54^.

It is essential to understand the historical processes and the relationships of power and domination that have permeated socioenvironmental conflicts in South America, mainly in terms of natural resources^4^; the need to satisfy urgent needs against the sacrifice of the natural patrimony, together with life quality conditions^55^. In fact, the hegemony associated to development and sustainability as strategies for progress^56^ circumscribes new forms of colonialism that make large-scale extractivism visible as the only way to attain economic growth^57^, and they are synonyms of territorial control in the guise of an ecological discourse that associates capital to the (use value/exchange value) of natural resources^58^.

Against this background, South America represents a threat for environmental activists and opponents to extractivism^59^. According to Global Witness, more than half of the murders of environmental activists recorded in 2018 (164 people) took place in the region^60^. 2019 showed an alarming increase in the number of murders (212 people), more than two thirds of which took place in South America. Mining continues to be associated to most of the murders, and the highest figure corresponds to Colombia (64 people)^61^.

The link between human rights, extractivism and the environment has given way to the recognition of intercultural perspectives in local and indigenous communities in terms of the relationship between the human and non-human world; consequently, it opens the road to new political settings for resistance based on human rights, governance of natural resources and the consolidation of social movements^62^. At the same time, participation mechanisms are strengthened by claiming the right to decide on extractive activities and on the type of development that must be attained in the regions^29^ for peasant groups, artisanal miners, ethnic groups, the urban population related with the place where extractive projects and tourist developments are underway^63^.

### Social representations theory

Social Representations Theory (SRT) originates in the concept of collective representations proposed by Durkheim in 1898^64^. It defines a social fact as the one that makes up the beliefs and trends of the collective group, as a result of common life and as a product of actions and reactions occurring in consciousness; therefore, it highlights the difference with collective consciousness^65^, which could only exist and manifest itself through individual consciousness^66^, considering they do not come from individuals taken in isolation^67^. In this sense, “collective representations translate the way in which a group thinks of itself in its relationships with the objects affecting them^65^”.

Moscovici proposed the SRT in the contemporary context of social psychology. He highlights that representations are being permanently constructed in a setting of interactions and actions. This posture differs from the one of the Durkheim school, where subjects find the pre-established representations without their intervention, they have a coercive nature and they are conformed without restriction^68^.

Social representations are almost tangible entities. They circulate ceaselessly in our day-to-day world, intersect and crystallize through a word, a gesture, an encounter. The largest part of close social relationships, of the objects produced or consumed, of the communications exchanged, are impregnated by them^69^.

Denise Jodelet identified three spheres to which SR belong, which facilitate their comprehension: subjectivity, inter-subjectivity and trans-subjectivity. The former states that people are not isolated; they are social stakeholders who become active through their participation in the network of interaction with others and their belonging to a social structure; the latter conceives representations as negotiated or commonly established elaborations resulting from dialogical exchange; the third one corresponds to the public and social setting in which representations circulate (mass media, imposed frameworks, ideological pressure and impositions)^70^.

SRT relates the cognitive process, social interaction and logics^71^. In this framework, the subject, as a generator of social settings, is influenced by mutual situations of communication and culture; hence, SR should be studied in specific contexts of the social sphere and current forms of life in order to have an individual vision of the surroundings and construction of reality^72,73^. In this way, SRT rejects the duality between individual and society based on the fact that the symbolic production is always the result of the interaction of the individual subject, the other, and the target world^74,75^.

## Method

The ethnographic design includes the analysis of social practices, beliefs, knowledge and guidelines of behaviour of the participants in the study^76^. It is understood as a process to acquire knowledge about a certain sociocultural reality^77^, when describing behaviours and beliefs of a group in everyday life^78^. A critical ethnographic design was considered, which aims to clarify the situation of the participants in terms of injustice, repression and inequality^79^, with the purpose of changing that reality thanks to the contributions of the study^80^.

The qualitative approach led us to understand meanings interpreted by the participants who make up the Santurban moorland^81^, based on their social, cultural and environmental characteristics. SR in a subjective and intersubjective level^68,70,82^ was taken as an analysis axis of the territory and of water as a vital resource.

### Population and sample

The population corresponds to inhabitants of the five municipalities mainly affected by the socioenvironmental conflict in the vicinity of the territory. According to the last population census from the National Administrative Department of Statistics [DANE]^83^, the figures by number of inhabitants in each municipality are as follows (Table 1):

**Table 1:** Number of inhabitants by municipality.

Province	Municipality	Population 2018
Santander	California	2130
	Suratá	3975
	Tona (Berlin village)	7373
	Vetas	2114
	Bucaramanga Metropolitan Area (Bucaramanga, Giron, Floridablanca, Piedecuesta)	1,204,093

The sample is made up by rural and urban populations. On the one hand, settlers of Vetas, California, Suratá and the village of Berlin (located in the municipality of Tona), whose economy has traditionally been based on ASM, agriculture and cattle farming^84^. From another angle, the inhabitants of the Bucaramanga Metropolitan Area (BMA) are included, as they get water from the Complex basins, from the sub-river basin of the Suratá river, served by the rivers Vetas, Charta and Tona. The settlers of that area are themselves affected by the quality and the amount of water provided downstream, because of ASM activities conducted in the higher moorland^85^.

A diverse and in chain sample was selected, in as much as we aimed to include a variety of perspectives for the issues studied (rural–urban) and to identify differences, similarities and patterns^79^. Initially, the municipalities bordering Santurban were visited to talk with leaders and groups who were participating in the process of boundary delimitation, organized by the national government. Regarding the BMA, meetings were held with people who make up the committees for the protection of water and the moorland. After establishing personal and telephone communication with different leaders, meetings were summoned and organized in each municipality to socialize the research project, its objectives and scope. Doubts were clarified and participants were encouraged to summon other peers who could contribute to the research thanks to their experience and knowledge.

In this way, 16 stakeholders were selected under the following criteria: availability of time, interest in the study, knowledge of the context of the socioenvironmental conflict, experience in ASM, participation in the boundary delimitation process and activism in the defence of water in the BMA. As a result of this selection, the stakeholders referred us to others who would be gradually included in the sample, until a considerable number of participants in the urban and rural environment was reached. The sample was made up by 176 participants, 118 of them from rural areas and 58 from the urban area. It is important to mention that informed consent was obtained from all participants (over 18) with the protocols approved by Committee on Ethics in Scientific Research of Universidad Industrial de Santander (CEINCI), the research group INNOTEC and the Educational Research Group Atenea. All methods were carried out in accordance with regulations from Colombian rules. Table 2 shows the number of people according to the association reference that added them to the sample.

**Table 2:** Sample according to association referent and the number of participants.

Association referent	Number of people
Members of Community Action Boards	36
Collective members for the defence of water and the moorland in the BMA	11
Representatives of artisan and low impact mining associations	5
ASM Miners	19
Inhabitants of the municipalities surrounding the moorland	58
BMA Settlers	47

### Data collection and analysis

The collection of information was made by means of structured focal groups of between 12 and 15 people. A facilitator from the research group was assigned to guide the questions, as well as a person to keep record of the focal group discussion^86^. Participant observation was implemented as a tool to learn about the daily life of the participants and to strengthen the SR analysis process, facilitating the interrelation between the researcher and the research subject^87^. Participants voluntarily filled out an informed consent at the time of participating in the focal groups. It is worth mentioning that the people in the sample asked not to leave any photographic nor audio-visual record of the moments when the information was collected.

A guide of ten questions was proposed for the development of the focal groups (Appendix 2), guided by two categories compatible with the study's objective: *territory* and *water as a vital resource*. The first one was discussed with the rural communities and the second one with the urban settlers, due to the identity features of each population segment and the differences between the two conflicting contexts. From the economic perspective, the rural component is associated to the territory that supports the activities of the primary sector, like the extraction of natural resources^88^; therefore, the relationship is constructed with respect to the territory as a means of survival. From the urban perspective, the goods of nature are perceived in relation to the interests of the people, as the water reaches the municipality through a network of services^89^; hence, the contamination of the environment in the urban surroundings shows a radical transformation in how the natural resources are perceived.

For the construction of the research instrument, the validation technique used was validation by expert judgement in order to adapt the cultural, technical and legal meanings of the subject of study. The reliability criterion was implemented to grant precision to the instrument and to discard conceptual or semantic errors^90^. Two professors and researchers from Universidad Industrial de Santander (UIS), with recognized expertise and knowledge on the socioenvironmental conflict of reference, contributed to the construction of the instrument with practical suggestions and observations on the subject. Both scholars were emphatic in establishing the two mentioned categories, in as much as each group (rural–urban) pursues tangible objectives based on the social and economic reality that they experience in their daily lives.

The first scholar clarified some legal concepts surrounding the boundaries delimitation process. Based on her experience, she suggested the importance of knowing the meanings participants hold with respect to the determination to delimit the moorland, to grant environmental licensing, the influence of the national government in the influence of the national government in the process and the importance of participation. Based on the experience of second scholar in the area of economics and socioenvironmental responsibility, he recommended to conduct research on the existence of sustainable practices for the protection of the desert and the water by miners; to further study the perceptions of BMA stakeholders on ASM, and to reflect on the meaning of teamwork to defend the water and the Santurban moorland, together with the rural communities that reside in the vicinity of the territory.

The NVIVO Qualitative Data Analysis Software was used, as it allowed us to encode information in an articulated, rigorous manner^91^. The subcategories emerged in an inductive way once the constant comparative method was applied, based on the search for similarities and differences through the analysis of the incidents collected in the data^92^. As reliability criterion, the codes or themes were defined according to the attributes and descriptors found in the two categories determined, in order to provide the members of the research team with a precise codification scheme for the analysis of transcripts, to ensure agreement among encoders and to standardize the text units^93^. Table 3 shows the categories matrix that emerged from the analysis process.

**Table 3:** Categories matrix.

Socioenvironmental Conflicts in the moorland ecosystem			
		Category	SR over the territory
Rural	Vetas, California, Suratá and Berlin district	Subcategories	Delimitation
			Mining
			Identity
			Ancestry
			Protection

		Category	SR over water as a vital resource
Urban	BMA	Subcategories	Delimitation
			Mining
			Vision of rurality
			Participation
			Environmental impact

To validate the results of the study, data triangulation was implemented, which should be understood as a process that is carried out to respond to a research problem; it consists of using different forms of data on a subject of study, with the purpose of contrasting different approaches to interpret them subjectively and intersubjectively^94^. In this case, the triangulation was carried out to guarantee the consistency of the results of the study, taking into account three aspects: first, the point of view of the theorists; second, the social representations of the moorland inhabitants on small and large-scale mining extractivismo, and the dynamics of socioenvironmental conflicts associated with mining activity; and third, the perspective of the research group^95^. Triangulation as a qualitative research technique refers to the comparison of the results of the three edges^96^, in order to build a vision which is closer to reality by identifying points of convergence or divergence to interpret a human phenomenon, from different angles, and thus ratify the validity of the findings^97^.

## Results

### Territory from the rural perspective

The meanings constructed by the rural settlers of Vetas, California, Suratá, and the Berlin village correspond to feelings of uneasiness regarding their immediate future, as they argue they are in a situation of uncertainty facing the impossibility to continue developing their ancestral survival activities (ASM, agriculture and cattle farming). This downfall arises within the framework of the probable extension of the delimitation scale for the Complex, in which case they would not be able to develop their daily economic activities.

For settlers, the delimitation process is not a synonym of the protection of the moorland ecosystem, as their being banished would mean the definite abandonment by the State towards a biodiverse ecosystem that has structurally been neglected in terms of financing and governance, even with the permanent presence of families in the area. Consequently, they think it is a priority to define the delimitation in order to defend their territory and protect their future from the imminent risk of disappearing from a natural patrimony they feel is theirs.

Our municipality of Suratá is one of the municipalities that is being more seriously affected by the delimitation of the moorland ecosystem, since approximately 9295 hectares are being compromised (Participant 38).

On the other hand, ASM have been their means of livelihood, and they gave up the use of mercury in the extraction of minerals as a guarantee of their care of water and the environment. Starting off from these principles and their experience, they demand the formalization and legalization of ASM, the verification of processes to exert responsible mining, prevent foreign involvement and design a special ancestral mining reserve.

They come here to ask: water or gold? Well, we tell them, water and gold (Participant 19).

Being able to show the world that, properly done, mining is not a synonym of damage or destruction (Participant 96).

As mentioned, ASM is part of the identity of settlers, and it is a lifestyle that frames the ancestry of an inherited activity, with a legacy of protection for the environment that comes before the economic interests and is linked with a commitment with care and the custody of the moorland ecosystem. Hence, the rural community feels offended by the stigmatization by city settlers, who blame them for polluting the water and destroying the moorland ecosystem.

We are proud to be ancestral miners and to be the real protectors of the moorland ecosystem (Participant 25).

It hurts to hear how they call us murderers because they say we are poisoning the water. We have been subjected to stigmatization, we have been made fun of, and sports settings have been closed to us just because we say we are from Santurban (Participant 102).

### Consumption and defense of the water

Contrary to the meanings assigned to the delimitation process from the rural area, urban settlers are interested in extending the demarcation scale of the moorland ecosystem at its most in order to protect a considerable area of the ecosystem; however, they overlook the adverse consequences that could deteriorate the quality of life in the rural area. For urban inhabitants, water as a vital resource is at the center of their actions for the defense of the ecosystem, as they assign to it a value of dependence, within the framework of the socioenvironmental conflict linked to climate change, disputes to save this resource, its scarcity and commercialization.

The delimitation of a moorland ecosystem cannot end up limiting the right to water by present and future generations (Participant 55).

In fact, they perceive mining as a threat to the consumption of the water that comes from the moorland basin, especially regarding large-scale mining as developed by multinational companies that persistently demand environmental licenses to conduct their exploration and exploitation operations; hence, urban settlers insist in annulling the extraction of gold and mining projects that lead to irreversible environmental impact, referring to “mega-ecocide” as the complete and uncontrolled destruction of the ecosystem.

The term mega-ecocide expresses how mega-mining projects in Santurban would lead to damage that we will not be able to mitigate or reverse for centuries ahead (Participant 49).

From the urban perspective, participation mechanisms have been essential to exert pressure by promoting the preservation of water, starting with social protest and popular consultation; however, citizens perceive that the relationships of power that are established in decision making have become a threat to those who defend the moorland ecosystem and the limited access to information restricts balance in the decisions.

They have faced reprisals on the exercise, recognition and materialization of their rights (Participant 5).

When social protest was felt in Bucaramanga, the State’s environmental agencies started to be concerned (Participant 22).

Urban settlers reiterate the importance of protecting and guaranteeing permanence in their lands by rural families located in the periphery of the moorland ecosystem, but their proposals have been scarce or nonexistent. On the one hand, one segment is consequent with the possible impact caused; on the other hand, lack of coherence and knowledge by some citizens on the effects of expanding the delimitation scale is clear, showing a perception of rurality limited to some socially constructed imaginaries which are far from the social, economic, cultural and environmental reality experienced in this context.

## Discussion and conclusions

The contrasting perspectives of rural inhabitants, who depend on the biodiversity provided in the moorland area to develop mining activities in their territories, and those of urban residents as consumers of the water coming from the Complex river basins are sharply divided. Within this framework, the difference between the rural–urban positions of the inhabitants of Vetas, California, Suratá, the Berlin village and the BMA indicate two premises: first, settlers recognize the existence of a socioenvironmental conflict and are conscious of their opposing interests. Second, a significant number of citizens ignore the definitive impacts on the countryside when demanding that the scales of boundary delimitation of Santurban be extended in order to protect a considerable segment of the ecosystem.

When interpreting the SR of rural and urban settlers within the framework of the socioenvironmental conflict characterized by the complexity of its social, economic, cultural and environmental components, it was possible to identify the meaning constructed by the stakeholders from their subjectivity when interacting with the territory and the environment^89,98^. Additionally, it was concluded that the appreciations that result from intersubjectivity in group exchanges are related to social identity and are transmitted in daily life by means of inherited traditions, expressions, emotions, knowledge and beliefs. This idea goes in line with the different arguments from scholars who have studied SR and the environmental-extractivist component (e. g.^99–101^).

However, the results show that TSR accentuates the bond SR-interindividual communication^102^, as SR provides meaning to the experience in a constructive way and determines the way in which individuals respond to that experience. Consequently, rural settlers identify themselves as a group when they enter an agreement and share the fact that delimitation may separate them from their territory and restrict their economic activities; likewise, they undertake the need to continue defending and protecting the moorland ecosystem as a mode of resistance linked to their convictions and legacy. Here lies the importance of SR as modalities of thought that guide the comprehension and command of their social, material and ideal surroundings^82^.

Ultimately, regarding the meanings, practices and values stakeholders hold over their territory and water as a vital resource, they are in line with the scope of two factors: the legalization of ASM and participation mechanisms in the process of environmental licensing of large-scale mining projects.

Extractivism triggers different forms of social impact that affect local communities^1^ and government bodies are influential, from structural factors to these triggers^5^; hence, governments with ASM areas should regulate the informality of a mining sector permeated by particular economic interests^103^ through solid regulations that tackle the possible benefits in terms of rent and work, also linked to environmental and social problems with significant impact in the groups and communities of influence^104,105^.

For artisanal miners in the municipalities of the Complex, formalizing mining in the region is essential. In addition to the SR presented throughout this study, formalization prevents the possible interference of armed groups that promote this activity in illegal settings, exacerbating the socioenvironmental conflict^106^. Although ASM in the region is a synonym of ancestry and survival, it is crucial to recognize the potential for environmental damage of gold processing^104^. In this sense, Pulido & Durán^107^ interpret that the SR of miners incorporate a discourse for the defense of the activity, including elements that reduce the threat felt by other stakeholders, referring to sustainable mining, employability and reactivation.

According to Rochlin^108^, failure in the formalization of ASM persists, due to the combination of a weak State, an insurgent organization of illegal industry and policies that generally exclude the interests of informal miners; added to preferences for the mega-mining industry. Consequently, it is necessary to promote certified practices for responsible mining that do not depend on public administration and are supported by Non-Governmental Organizations, as in the case of the initiative Oro Verde in Choco (Colombia), which involves strict ecological standards^21^. This practice shows the disposition of miners to know and to share the best practices in environmental management, thinking of future generations^109^; added to the possibilities to create income as an alternative for survival, and to share it with the local economy^104^.

The participants who make up the artisan and low-impact mining associations are traditionally made up by families and they have tried to legally organize the gold mining activities at the Complex. Although high impact mining has a significant influence in the province of Santander, miners propose financial and technological assistance which, in agreement with Hilson^110^, should start with a change of narratives with respect to the ASM sector and its operators, especially in the design of environmental management plans that start with from formalization^111^. These elements would allow economic competitiveness to be sustained and environmental controls to be implemented, through the accompaniment of the national government and the final decisions taken in the delimitation process with the communities.

In parallel, stakeholders have had significant experiences of citizen participation regarding the nullity or suspension of mining projects that in the past decade have involved foreign interests of large-scale mining exploitation. According to Arce et al.^112^, people who perceive their government has the capacity to respond to their claims are ready to protest; conversely, those who observe the incapacity to establish long-term changes doubt the effectiveness of exercising this right. Hence the importance of exchanging information between the civil society and the regulating state in the dynamics of a socioenvironmental conflict, due to the unequal conditions to access institutional mechanisms, to look for allies and to model strategies to obtain results^113^.

Argentina has been a pioneer in community participation facing mega-mining projects. Self-Organized Neighbors Assemblies have promoted several consultations within the framework of socioenvironmental con, especially the one developed in Esquel (second popular consultation conducted in South America), since neither an environmental impact study nor a public hearing were held prior to exploration activities; hence, the people focused on promoting decision-making settings. This social resistance resulted in rejection of the mining exploitation by 81% of the population in 2003, and shortly thereafter, the laws were sanctioned to prohibit this activity in eight provinces^15,16,114,115^.

It is essential that citizens become active for the protection of water as a non-renewable resource^14^, joining efforts to mitigate the expansion of extractivism and to limit access to natural resources^116^; likewise, they take precedence over power relationships that question political and administrative institutions^117^, in the South American context, characterized by an unbalance in the negotiations between the community and the extractive industry^5^.

Indeed, the socioenvironmental conflict presented in this study requires the ongoing participation of rural and urban settlers, a factor that was made evident in the development of focal groups and participant observation; in consideration to their permanent questions on the current reality in their regions and the permanent search for solutions, which sometimes seems to depend on subjective SR from the immediate surroundings. Consequently, settings for participation and agreement must be granted in order to eliminate threatening trends at the time of implementing actions for the defence of their territory and natural resources.

## Supplementary Information

Supplementary Information 1.

Supplementary Information 2.
