# Supplementary material for: Socioenvironmental conflicts and social representations surrounding mining extractivism at Santurban
Source: Sci Rep. 2022 Jun 15;12:9948. doi: 10.1038/s41598-022-14086-0 (PMC9200705; doi:10.1038/s41598-022-14086-0)
Supplement: Supplementary file 1 — Supplementary Information 1. [file 41598_2022_14086_MOESM1_ESM.docx]

**Appendix 1**

**Glossary of Acronyms**

Sociedad Minera de Santander (Minesa)

Ministry of Environment and Sustainable Development (MADS)

Artisanal and Small-scale Mining (ASM)

Social Representations (SR)

Social Representations Theory (SRT)

National Administrative Department of Statistics (DANE)

Bucaramanga Metropolitan Area (BMA)

Universidad Industrial de Santander (UIS)
