# Supplementary material for: Socioenvironmental conflicts and social representations surrounding mining extractivism at Santurban
Source: Sci Rep. 2022 Jun 15;12:9948. doi: 10.1038/s41598-022-14086-0 (PMC9200705; doi:10.1038/s41598-022-14086-0)
Supplement: Supplementary file 2 — Supplementary Information 2. [file 41598_2022_14086_MOESM2_ESM.docx]

**Appendix 2**

**Guiding questions for focus groups**

**Territory**

1. What are the reasons that have triggered socioenvironmental conflicts regarding the delimitation of the Santurban moorland?

2. What do you think about ASM?

3. What does the Complex of Jurisdiction-Santurban-Berlin moorland ecosystem territory mean to you?

4. How do you perceive the environmental impact resulting from anthropogenic activities developed within the moorland ecosystem?

5. How have you participated in the process of boundary delimitation of Santurban?

6. How do you consider the process of boundary delimitation of Santurban should be conducted?

7. What is the connection between the ASM and the environment?

8. What is your perception on the environmental licensing in this type of protected areas?

9. What role should authorities play in regulating the mining activity?

10. What guarantees should the national government give to the communities living in the municipalities bordering the Santurban moorland, with respect to the development of ASM as the main economic activity in the region?

11. How do you think the population of the BMA perceives you, in terms of the economic activity that you develop and the defence of this activity in environs of Santurban?

**Water as a vital resource**

1. What does water mean for you as a vital resource?

2. What does ASM mean to you with respect to the protection of the water and the moorland ecosystem?

3. What is the connection between Human Rights and the boundary delimitation of the moorland?

4. Which is the responsibility of the national government regarding the preservation and protection of the water and the moorland ecosystem?

5. How do you perceive the development of large-scale and ASM in the vicinity of the moorland ecosystem?

6. What is your perception on the community that develops the ASM activity in Santurban moorland?

7. Which are the responsibilities and roles of urban communities to preserve and to protect the water and the moorland ecosystem?

8. Do you consider it plausible to work together with the rural community to protect the moorland? If so, which would be the commonalities and controversial points?

9. In your opinion, how should the process of boundary delimitation of Santurban moorland be conducted?

10. How should citizen participation be exerted in the process of boundary delimitation of the moorland?

11. What is your perception on the environmental licensing in this type of protected areas?

12. In the event that ASM activities continue to be developed in the region, which are the guarantees demanded by the BMA population with respect to the quality of the water that comes from the moorland and is consumed daily?
